# Development of a metric for tracking and comparing population health based on the minimal generic set of domains of functioning and health

**DOI:** 10.1186/s12963-016-0088-y

**Published:** 2016-05-12

**Authors:** Cornelia Oberhauser, Somnath Chatterji, Carla Sabariego, Alarcos Cieza

**Affiliations:** Department of Medical Informatics, Biometry and Epidemiology—IBE, Chair for Public Health and Health Services Research, Research Unit for Biopsychosocial Health, LMU Munich, Munich, Germany; Faculty of Social and Human Sciences, School of Psychology, University of Southampton, Southampton, UK; Swiss Paraplegic Research, Nottwil, Switzerland; Surveys, Measurement and Analysis, Department of Measurement and Health Information Systems, World Health Organization, Geneva, Switzerland

**Keywords:** Minimal generic set, Functioning, Health, Item Response Theory, Partial Credit Model, Health metric, Psychometric properties, Internal consistency reliability, Construct validity, Sensitivity to change

## Abstract

**Background:**

The following minimal set of valid health domains for tracking the health of both clinical and general populations has recently been proposed: 1) energy and drive functions, 2) emotional functions, 3) sensation of pain, 4) carrying out daily routine, 5) walking and moving around, and 6) remunerative employment. This study investigates whether these domains can be integrated into a sound psychometric measure to adequately assess, compare, and monitor the health of populations.

**Methods:**

Data from waves 3 and 4 of the English Longitudinal Study of Ageing (ELSA) were analysed (*N* = 9779 and 11,050). From ELSA, 12 items operationalizing the six domains of the minimal generic set were identified. The Partial Credit Model (PCM) was applied to create a health metric based on these items. The Item Response Theory (IRT) model assumptions of unidimensionality, local independence, and monotonicity were evaluated, and Differential Item Functioning (DIF) was examined for sex and age groups. The psychometric properties of: 1) internal consistency reliability, 2) construct validity, and 3) sensitivity to change were evaluated to establish the final health metric.

**Results:**

IRT model assumptions were found to be fulfilled. None of the items showed DIF by sex or age group. The final health metric demonstrated sound psychometric properties.

**Conclusions:**

The health metric developed in this study – based on the domains of the minimal generic set – proved useful for a wide range of health comparisons, especially for different groups of persons, and both cross-sectionally and over time. Monitoring health over time provides especially useful information for health care providers and health policymakers and both in clinical settings and the general population. The developed health metric offers a wide range of applications, including comparisons of levels of health among different groups in the general population, clinical populations, and even populations within and across different countries.

**Electronic supplementary material:**

The online version of this article (doi:10.1186/s12963-016-0088-y) contains supplementary material, which is available to authorized users.

## Background

Measuring the health of populations in a conceptually and cross-culturally valid manner is important from different perspectives. From the point of view of epidemiologists, sound measurement of health is essential both to estimate the overall burden of ill health in specific populations and to compare the relative impact of specific health problems across different groups of populations at risk [1]. From the point of view of policymakers, it is essential to monitor the effectiveness of health care [2] and, generally, to provide evidence for setting goals, implementing, and monitoring public health policy [3].

One frequently used approach to measure the health of populations is to generate a composite score of overall health, taking into consideration disease severity in terms of the impact of health conditions on individuals. In this approach, a set of meaningful domains of functioning, such as walking, self-care, memory, and pain, is selected and used to produce a score. This approach, however, does not account for comparability per se. If the domains of functioning included in studies and surveys and the method of creating a corresponding composite score vary largely, comparability is compromised.

A recently published psychometric study addresses this challenge by using the International Classification of Functioning, Disability and Health (ICF) [4]. A universally valid minimal set of domains of functioning to track the health of both clinical and general populations was proposed [5]. This “minimal generic set of domains of functioning and health” includes six ICF domains: 1) energy and drive functions, 2) emotional functions, 3) sensation of pain, 4) carrying out daily routine, 5) mobility (including walking and moving around), and 6) remunerative employment. These domains from the minimal generic set are closely related to the World Health Survey (WHS) domains, which in addition contain cognition and vision [6, 7]. However, while the WHS domains were developed to capture population health states and to quantify health using the most parsimonious set of domains that also intuitively match individual notions of health and are used in general population survey instruments, there have been no attempts outside of WHO to investigate the relevance of this set of domains across different populations, and, in particular, in the clinical population [5]. In contrast, the minimal generic set was developed taking both data from the general population and from clinical populations into account [5].

This selection of domains does not constitute an instrument and these domains can be operationalized in very different ways in different studies. Therefore, our approach can be applied, a posteriori, to already existing data in an ex post harmonization exercise across multiple data sources. Additionally, the inclusion of such a generic set of domains in other data sources such as electronic health records, and when overlapped with population health surveys that contain these domains, could allow the quantification of health in a given population that spans the full spectrum from community dwelling to patient populations. In comparison to WHODAS 2.0, an instrument developed by WHO applicable in both clinical and general population settings, the minimal generic set does not only focus on activities and participation domains, but also contains body functions [8].

The authors understand this set as a starting point to address the important challenge of achieving comparability of data across studies and countries in health measurement. They stress the necessity to confirm whether this set can be used to develop a health metric useful for assessing and comparing trends in population health [5].

Furthermore, this generic set is very brief and made up of domains of functioning addressed in questions posed in most health and disability surveys, including surveys in which no standardized questionnaire, like WHODAS 2.0, has been used. It demonstrates that a psychometrically sound metric with cardinal properties can be constructed to monitor a health status over time using existing data. A metric with cardinal properties does not only register the presence or absence of change, but also quantifies the extent of that change. Health inequalities and their extent could also be identified, as well as the effects of non-fatal health outcomes on overall population health by aggregating individual levels over the population.

This study aims to investigate whether data on the domains of the minimal generic set can be integrated into a psychometrically sound health metric. The specific aims are to evaluate the 1) internal consistency reliability, 2) construct validity, and 3) sensitivity to change of this metric.

## Methods

### Data

Data from the English Longitudinal Study of Ageing (ELSA) was used for the analysis. ELSA is a biannual, longitudinal, and nationally representative survey that focuses on adults aged 50 and over living in private households in England [9]. More concretely, data from waves 3 and 4 collected in 2006–2007 and 2008–2009 were analysed (*N* = 9779 and 11,050). Depending on the requirements for each specific aim, the combined data, wave-4 data, or the overlapping data (i.e., persons whose data was available for both waves 3 and 4) were used.

From the ELSA survey, 22 questions were identified that operationalize the six domains of the minimal generic set as presented in Additional file 1. For energy and drive functions, emotional functions, walking and moving around, and remunerative employment, the questions directly reflected the content of the respective domains. Therefore, the original variables were used in the analysis. For sensation of pain, the first question (“often troubled with pain”) served as a filter for the second (“severity of the pain”), which means that the second question was only asked if the first was answered “yes.” Therefore, these two questions were summarized into one item with response options “not often troubled with pain,” “mild,” “moderate,” and “severe” pain.

A different strategy was needed for the domain “carrying out daily routine,” as the individual questions as framed in the survey did not reflect that domain. Therefore, two sum scores were created. One addresses activities of daily living (ADLs), including dressing, washing, eating, getting in and out of bed, and using the toilet, with values ranging from none to five limitations. The other score sums up instrumental activities of daily living (IADLs), including difficulty using a map, preparing a hot meal, shopping for groceries, taking medications, doing housework, and managing money, with values ranging from none to six limitations.

The response options for these selected items were coded or recoded with higher values consistently reflecting worse health.

### Analysis

#### Descriptive statistics

Descriptive statistics were used to characterize the study population.

#### Development of the health metric

The Partial Credit Model (PCM) was applied to develop a health metric [10, 11]. The PCM or Polytomous Rasch Model is a unidimensional Item Response Theory (IRT) model that can be applied to a set of ordinal, polytomous items [12]. Based on the model, a latent scale is defined on which both persons and items can be located. For persons, their location is called “person ability,” while for items, the terms “item location” or “item difficulty” are used. In addition, item thresholds which indicate the locations on the latent trait where the item best discriminates between persons are available for each item.

Before the PCM was applied, the model assumptions evaluated were: unidimensionality, local independency, and monotonicity.

Unidimensionality [13] was evaluated with bifactor analysis [14–16], which assumes the presence of a single general factor and multiple independent group factors. If all items load high on the general factor score and the factor loadings on the general factor score exceed those of the group factors, an underlying unidimensional latent trait can be assumed. The number of factors considered in the bifactor analysis was determined based on permuted parallel analysis [17]. Bifactor analysis was applied on the polychoric correlation matrix [18, 19], which constitutes a measure of association between two latent continuous variables underlying two measured ordinal variables.

Local independence [13] was examined based on the residual correlations among items resulting from a single-factor factor analysis [20]. The PCM was then estimated with and without the flagged possible local dependent items (residual correlations higher than 0.2) to see if results were robust to questions’ dependencies [21]. If the item thresholds fundamentally change when considering local dependent items in the same model, all but one of them must be excluded.

Monotonicity was studied for each item by examining graphs of the item’s distribution conditional on mean “rest-scores” [13] calculated for each person as the average raw score of all the remaining non-missing items. If there is a consistent trend that persons with higher mean “rest-scores” are more likely to have more problems in the selected item, monotonicity can be assumed. Items violating the monotonicity assumption need to be excluded from the model.

After the evaluation of model assumptions, the PCM was fitted. If thresholds were unordered, the response options of the affected items were collapsed until all thresholds were in the correct order.

To examine whether persons from different groups with the same (latent) health level have a different probability of giving a certain response to an item, differential item functioning (DIF) was tested for gender and age groups (< = 64 and >64) using iterative hybrid ordinal logistic regression with change in McFadden’s pseudo R-squared measure (above 0.02) as a DIF criterion [22, 23]. Items showing DIF must be split into two separate items for the two groups and the model is re-estimated.

To examine whether the items fitted the PCM, (unweighted) outfit and (weighted) infit mean squares were calculated [24]. Values close to 1 indicate good item fit, while values “much” larger than 1 indicate underfit (i.e., the observed data varies much more than can be explained by the model, constituting a violation to the model). Values “much” smaller than 1 indicate overfit (i.e., the data varies much less than would be expected based on the model, what is usually considered acceptable) [11, 24]. In the literature, a range from 0.7 to 1.3 is generally accepted [24]. However, to better interpret these mean square statistics, the expected probabilities for responding above a certain threshold were graphically compared to the observed response frequencies for groups of persons with close ability estimates.

Finally, persons’ health levels were linearly transformed into a health metric ranging from 0 (worst health) to 100 (best health), which facilitates judging the relevance of differences between groups, e.g., with and without health conditions and change over time.

According to the specific aims of this study, for the final health metric the following psychometric properties were evaluated: 1) internal consistency reliability, 2) construct validity, and 3) sensitivity to change.

Internal consistency reliability was assessed based on different measures. For each item, the inter-item correlation and the item-to-total correlation [25] were calculated using polychoric correlations. Cronbach’s alpha [26], McDonald’s omega hierarchical, and McDonald’s omega total [27] were used for the complete item set. All these measures can range from 0 to 1, with higher values indicating higher reliability.

Construct validity was assessed for wave-4 data based on four different criteria [25]:

Convergent validity was analyzed based on the Spearman correlation of the health metric with other health-related variables, like the general health question and a question on long-standing limiting illnesses. A high correlation indicates high convergent validity. Discriminant validity was analysed based on the Spearman correlation of the health metric with variables, like life satisfaction, the number of falls within the last year and age that are not direct assessments of health levels. A low correlation indicates high discriminant validity.

Concurrent validity was assessed based on a linear additive model [28] which predicts the value on the health metric based on sex, age, education, income, and health conditions as independent variables. Age is modeled in a flexible, non-parametric way using P-splines. Concurrent validity can be judged as high if persons with health conditions have a lower predicted level of health compared to those without the respective health conditions and persons with severe health conditions on average have lower predicted health levels than those with very mild health conditions. To objectively judge the severity of the prototypical health conditions, the disability weights estimated for 220 unique health states within the Global Burden of Disease Study 2010 (GBD 2010) [29] served as a reference.

Predictive validity was analyzed based on predicting mortality from 2008 to 2012. For this purpose, four additive logit-models [28] were compared, each containing the covariates sex, age, education, income, and health conditions (as above). Model 1 contains only these independent variables. Model 2 also contains the health metric, while model 3 additionally contains the general-health question. Model 4 contains all the covariates, as well as the general health question and the health metric. Age and the health metric are modeled in a flexible, non-parametric way using P-splines. For all these models, different model-fit criteria are compared based on: the adjusted R-square, the percentage of explained deviance, and the Akaike information criterion (AIC) [28]. If the inclusion of the health metric improves model fit, this indicates predictive validity.

Sensitivity to change was evaluated for the subsample on which data were available for both wave 3 and 4. A linear additive model [28] was fitted with the value of the health metric in wave 4 as a dependent variable and incidence of health conditions since wave 3 as independent variables, while controlling for the value of the health metric in wave 3 and additional covariates. If the incidence of severe health conditions has a high negative impact on the expected value of the health metric while the incidence of less severe health conditions has a smaller effect, the health metric shows high sensitivity to change.

All analyses were performed with R version 2.15.2 [30].

## Results

### Descriptive statistics

Descriptive statistics of the study population are provided in Table 1.

**Table 1:** Descriptive statistics of wave-3 and wave-4 data and their overlap

	Wave 3	Wave 4	Overlap of wave 3 and 4 - wave 4 values
	(*N* = 9779)	(*N* = 11,050)	(*N* = 7908)
Age: mean (median)	64.56 (63)	65.24 (64)	66.40 (65)
Gender: female (%)	0.56	0.55	0.57
Education: low (%) ^a^	–	0.42	0.42
Education: medium (%) ^a^	–	0.27	0.27
Education: high (%) ^a^	–	0.31	0.31
Income: low (%) ^b^	–	0.31	0.32
Income: medium (%) ^b^	–	0.33	0.33
Income: high (%) ^b^	–	0.36	0.35
General health ^c^			
w3: very good/w4: excellent (%)	0.26	0.13	0.12
w3: good/w4: very good (%)	0.43	0.29	0.29
w3: fair/w4: good (%)	0.24	0.32	0.33
w3: bad/w4: fair (%)	0.06	0.19	0.19
w3: very bad/w4: poor (%)	0.01	0.07	0.07

^a^ The education division is from a level lower than “O-level” or equivalent (typically 0–11 years of schooling), qualified to a level lower than “A-level” or equivalent (typically 12–13 years), and a higher qualification (typically >13 years)

^b^ Income groups were formed by dividing equalised total income into three approximately equally sized groups based on the sample

^c^ The response options for the general health question differed for the two waves, leading to a very different response pattern. “w3” and “w4” are abbreviations for wave 3 and wave 4, respectively

^–^ Information on education and income was incomplete for wave-3 data and is, therefore, not included in the table

### Development of the health metric

When testing the IRT model assumptions for the combined dataset, permuted parallel analysis indicated the presence of two factors. In the bifactor analysis, the general factor accounted for 50.8 % of the variance. Its factor loadings (ranging from 0.55 to 0.85) exceeded those of the group factors for all items, supporting the assumption of unidimensionality. High residual correlations were found for “feeling everything was an effort” and “could not get going” in the domain “energy and drive functions” and for feeling “depressed,” “sad,” or being “happy” in “emotional functions.” When keeping only one of the local dependent variables for each domain (“feeling everything was an effort” and “depressed”), sensitivity analyses showed similar thresholds compared to the model with all items included (Pearson correlation of 0.99), which indicates that all items can be kept in the final model. Monotonicity was graphically confirmed for all items.

When fitting the PCM onto the combined dataset, the thresholds of four items were disordered and had to be collapsed: for “pain” and “walking a quarter of a mile,” “mild” and “moderate” problems were collapsed. For the two scores on ADL and IADL, the response options were collapsed into “no limitation,” “one or two limitations,” and “three or more limitations.” None of the items showed DIF by gender or age based on the selected DIF criterion.

All items reasonably fitted the PCM. As presented in Additional file 2, the outfit and infit mean squares were rather close to one for most of the items. Furthermore, the curves in Additional file 3 confirm that the observed response frequencies (for responding above a certain threshold) for groups of persons with close ability estimates are quite close to the expected probabilities, especially for items with mean square statistics further away from 1. In most cases, the curve of observed frequencies is steeper, which corresponds to the definition of overfit.

**Fig. 1:**
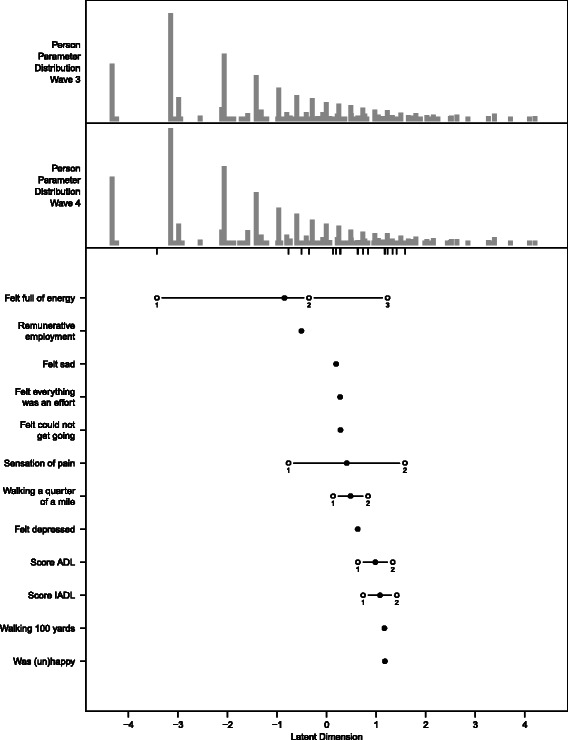
Person-item map for the final PCM. The top part displays the distribution of persons’ health levels separately for wave 3 and wave 4. The bottom part shows the item locations (*bullets*) and item thresholds (*circles*) for the 12 items. To facilitate the comparison of item thresholds with persons’ abilities, the item thresholds are additionally plotted below the persons’ distributions (of wave 4) by small vertical lines

The results for the final PCM are visualized in the person-item map in Fig. 1. In the top part of the figure, the distribution of persons’ health levels is shown separately for wave 3 and wave 4. The pattern of persons’ levels is quite similar in the two waves, with values ranging from −4.33 to 4.21. Item locations and item thresholds are presented in the bottom part of the figure. Item locations range from −0.85 to 1.17, while item thresholds range from −3.43 to 1.58.

The items are well suited to differentiate between persons’ levels in the medium range of health. They do not, however, differentiate well between the large proportion of very healthy persons (to the left) and the small proportion of extremely unhealthy persons (to the right).

### Internal consistency reliability

Table 2 shows the results of internal consistency reliability. The values of the different measures yield consistent results when calculated for each of the two waves separately and for the combined dataset. Inter-item correlation is high, but has high variability. Item-to-total correlation is higher with less variation. Cronbach’s alpha and McDonald’s omega total are quite close to 1. McDonald’s omega hierarchical is lower (with values around 0.60), but of reasonable size [27]. Therefore, all values indicate high internal consistency reliability.

**Table 2:** Results on internal consistency reliability

Measure	Wave 3	Wave 4	Waves 3 and 4 combined
Inter-item correlation: mean [min; max]	0.54 [0.23; 0.90]	0.53 [0.24; 0.92]	0.53 [0.25; 0.91]
Item-to-total correlation: mean [min; max]	0.76 [0.61; 0.84]	0.75 [0.59; 0.85]	0.75 [0.60; 0.84]
Cronbach’s alpha	0.93	0.93	0.93
McDonald’s omega hierarchical	0.60	0.61	0.61
McDonald’s omega total	0.96	0.95	0.96

### Construct validity

The Spearman correlation of the health metric with general health and long-standing illness is comparably high (−0.64 and −0.59), indicating high convergent validity. The Spearman correlation of the health metric with life satisfaction is lower (−0.36) and lowest for the number of falls (−0.25) and age (−0.23). These low correlations with the health metric indicate high discriminant validity since these items are not a direct measure of health status. Boxplots visualizing the relationship of the health metric with the five above-mentioned variables are presented in Additional file 4. The complete correlation matrix is presented in Additional file 5.

**Fig. 2:**
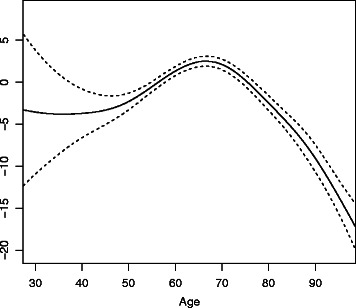
Results on concurrent validity – nonlinear effect of age. The nonlinear effect of age (solid line) and 95 % confidence intervals (dashed lines) resulting from the linear additive model predicting the value of the health metric for wave-4 data. As there are only a small number of observations below the age of 50, there is a lot of uncertainty in the estimation in this range

The linear additive model with the health metric as a dependent variable and sex, age, education, income, and health conditions as independent variables indicates high concurrent validity. Detailed results are presented in Table 3 and Fig. 2. As expected, all the listed health conditions have a negative effect on the health metric, with more severe health conditions, such as lung disease, arthritis, heart failure, Parkinson's disease, and dementia, showing a larger negative effect. This ranking is, where comparison is possible, in agreement with the disability weights estimated for 220 unique health states used in GBD 2010 [29].

**Table 3:** Results on concurrent validity – linear additive model predicting the value of the health metric

	Number	Coefficient	SE	*p*-value
Intercept		73.61	0.44	<0.0001
Gender (female)		−0.56	0.34	0.0965
Education (middle)		3.47	0.41	<0.0001
Education (high)		4.67	0.41	<0.0001
Income (middle)		2.08	0.40	<0.0001
Income (high)		5.43	0.42	<0.0001
Health conditions:				
High cholesterol	3546	−0.58	0.36	0.1108
Heart attack	741	−1.25	0.86	0.1459
Heart murmur	423	−1.26	0.84	0.1336
High blood pressure	4214	−2.44	0.35	<0.0001
Abnormal heart rhythm	820	−2.97	0.63	<0.0001
Angina	885	−3.31	0.80	<0.0001
Asthma	1260	−3.35	0.51	<0.0001
Cancer	571	−3.45	0.72	<0.0001
Other heart disease	303	−3.97	1.02	<0.0001
Diabetes	1063	−6.36	0.56	<0.0001
Osteoporosis	753	−7.46	0.65	<0.0001
Stroke	481	−8.44	0.81	<0.0001
Psychiatric condition	971	−9.92	0.57	<0.0001
Lung disease	544	−11.06	0.76	<0.0001
Arthritis	3816	−11.09	0.35	<0.0001
Heart failure	65	−12.38	2.17	<0.0001
Parkinson’s disease	79	−19.20	1.98	<0.0001
Dementia	154	−19.44	1.65	<0.0001

Regression coefficients, standard errors (SE), and *p*-values resulting from the linear additive model predicting the value of the health metric for wave-4 data. For the health conditions, the number of cases with the respective health condition is additionally provided. Health conditions are sorted by increasing effect. The nonlinear effect of age is shown in Fig. 2

The reference categories are male, low education, low income, and not having the respective health condition

Figure 2 shows the nonlinear effect of age on the health metric together with its 95 % confidence intervals. From an age of 68 on, age has an increasingly negative impact on health levels.

### Predictive validity

Table 4 shows the results regarding the predictive validity of the health metric. For wave-4 data, four different additive logit models predicting mortality are compared based on three model-fit criteria. When comparing the four models, all three criteria indicate higher model fit for those models with the health metric included (higher adjusted R-square, higher percentage of deviance explained, smaller AIC). This means that the health metric has a higher predictive validity for mortality than the general health question and that it still has predictive validity when added to the general health question.

**Table 4:** Results on predictive validity – comparison of model-fit criteria for four different models

	Adjusted R-square	Percentage of deviance explained	AIC
Model 1 including only covariates	15.2	23.6	3362
Model 2 including covariates and the health metric	17.5	26.2	3251
Model 3 including covariates and the general health question	16.7	25.5	3285
Model 4 including covariates, the general health question and the health metric	17.9	26.6	3240

For wave-4 data, four different additive logit-models predicting mortality in 2008 to 2012 are compared based on three model-fit criteria: adjusted R-square, percentage of deviance explained, and Akaike Information Criterion (AIC). Covariates considered in all four models include sex, age, education, income, and health conditions. To permit a fair comparison of criteria, the same subset of data with complete responses in all the variables considered over the four models was used. Where included, age and the health metric are modeled in a flexible, non-parametric way using P-splines

### Sensitivity to change

Table 5 and Additional file 6 show the results from the linear additive model predicting the value of the health metric in wave 4 based on the incidence of health conditions within the last two years when controlling for the value of the health metric in wave 3 and other covariates. Table 5 shows that the incidence of any of these health conditions has a negative effect on the value of the health metric. The incidence of severe health conditions, often without effective therapy and progressing rapidly, have a large negative effect, while incidence of mild health conditions or those with effective therapy have a smaller negative impact. Therefore, these results indicate high sensitivity to change. Additional file 6 shows the nonlinear effect of age from this model. However, age was only used as a control variable here.

**Table 5:** Results on sensitivity to change – linear additive model predicting the value of the health metric

	Number	Coefficient	SE	*p*-value
Intercept		18.98	0.65	<0.0001
Health metric in wave 3		0.71	0.01	<0.0001
Gender (female)		−0.86	0.30	0.0047
Education (middle)		1.42	0.38	0.0002
Education (high)		1.22	0.38	0.0012
Income (middle)		0.86	0.37	0.0200
Income (high)		1.79	0.39	<0.0001
Incidence of:				
High cholesterol	503	−0.57	0.61	0.3563
Angina	180	−0.73	1.00	0.4655
Heart attack	265	−0.94	0.83	0.2571
Osteoporosis	121	−1.17	1.21	0.3323
High blood pressure	325	−1.46	0.75	0.0502
Other heart disease	126	−1.94	1.19	0.1019
Abnormal heart rhythm	137	−2.27	1.15	0.0481
Asthma	91	−2.72	1,40	0.0514
Diabetes	138	−2.95	1.13	0.0093
Heart murmur	57	−3.06	1.73	0.0771
Arthritis	361	−4.06	0.71	<0.0001
Cancer	138	−4.15	1.12	0.0002
Stroke	91	−4.73	1.41	0.0008
Lung disease	91	−5.90	1.39	<0.0001
Parkinson’s disease	13	−6.51	3.56	0.0679
Psychiatric condition	110	−8.00	1.29	<0.0001
Heart failure	14	−9.62	3.90	0.0138
Dementia	66	−16.62	1.96	<0.0001

Regression coefficients, standard errors (SE), and *p*-values resulting from the linear additive model predicting the value of the health metric in wave 4 based on the incidence of health conditions within the last two years and controlled for the value of the health metric in wave 3 and other covariates. For the health conditions, the number of cases with incidence in the last two years is provided. Health conditions are sorted by increasing effect. The nonlinear effect of age is shown in Additional file 6

The reference categories are male, low education, low income and no incidence of the respective health condition within the last two years

## Discussion

In this study, we demonstrated that it is possible to develop a psychometrically sound metric useful to track and compare population health based on a recently proposed minimal generic set of domains of functioning valid in both the clinical and the general populations. This metric showed high internal consistency reliability, high construct validity, and high sensitivity to change. This metric of health has a huge potential, as it contains health domains usually included in health surveys currently conducted all over the world, which means that our approach can be applied, a posteriori, to many already existing surveys. Therefore, such a metric enables health comparisons even for studies in which no standardized instrument, like WHODAS 2.0, was, a priori, included.

The applicability of the PCM on the data was examined in a rigorous manner. All three model assumptions of IRT analysis, i.e., unidimensionality, local independency, and monotonicity, were formally investigated and found to be fulfilled. None of the items showed DIF by gender or age groups, and all items reasonably fitted the PCM.

The developed metric differentiated well between persons in the medium range of health, but less precisely at the lower and upper ends. Its creation constitutes a trade-off between two conflicting aims: on the one hand, maximal measurement precision is desired to permit fine distinctions in persons’ health levels and to increase its potential uses, both for group comparisons and comparisons over time. On the other hand, the metric should be based on an extremely parsimonious set of domains that can be integrated at low cost, both with regards to financial resources and (interviewing) time, into any already existing or newly developed survey or questionnaire. Only if the metric is based on a frugal set of domains will its domains be frequently implemented in studies. This, in turn, constitutes the prerequisite for the subsequent application of the metric for comparison purposes. Therefore, loss in measurement precision, especially at the margins of the continuum, must be accepted for reasons of practicability. Also from a public health perspective, a good differentiation in the middle range of the continuum gains special relevance since persons in that range of health are at risk of further deterioration, while little change in health state is expected for those at the margins – which does not mean that information on the margins is not relevant for policymakers. However, this does not imply that only questions addressing the six domains of the generic set should be used in every study. For specific purposes, e.g., in clinical studies, additional questions addressing other domains can be added, as has been previously proposed [5].

In addition to the validity criteria already mentioned, the validity of the metric is supported by further findings. As can be seen from the linear additive model to evaluate concurrent validity (Table 3), all of the well-known gradients of health – age, education, and income levels – are captured by the health metric [31]. The well-documented gender differences in health are also captured by the metric [31, 32]. Women are slightly worse off than men, even when several covariates are taken into account.

The health metric makes it possible to compare the health of populations at the same point in time and over a time period. Subgroups of the sample can be compared based on the results of the linear additive model used to examine concurrent validity. For example, persons with psychiatric conditions are expected to have about 10 fewer points on the metric, i.e., worse health than persons without psychiatric conditions when assuming that all other characteristics are the same. The distribution of health levels for wave 3 and wave 4 samples is visualized in the top part of the person-item map (Fig. 1). Furthermore, in the linear additive model used to examine sensitivity to change, the health status in wave 3 is used to explain the health status in wave 4, taking the incidence of specific health conditions into account.

In this study, the ELSA data was used for several reasons. It contained questions to operationalize all six domains from the minimal generic set. In addition to the questions on functioning, it contained information on socio-economic status, health-related variables and detailed information on health conditions. Finally, longitudinal data were available, which permitted the creation of the health metric for two waves, making comparisons over time possible. Because of all these properties, the ELSA data enabled all the analyses necessary to examine the psychometric properties of the developed health metric. From our experience with this longitudinal cohort study, we assume that similar analyses can be carried out in a straightforward manner with further existing surveys and studies.

There are some limitations of this study. Only one exemplary dataset, ELSA, was analyzed. England is a high-resource Western country and not representative of the general population worldwide. In addition, the focus of ELSA was on persons aged 50 and above and not on the general population without age restrictions. As the analyses were restricted to a single data source, the investigated set of questions was limited. But proposing questions is beyond the scope of the study. If slightly different content is asked in the questions or different response options are used, results might differ. Therefore, the results shown need to be validated in further studies using data from different populations with regards to country, age group, and setting. Additionally, as shown in our analyses, the set of items included in this metric does not allow discriminations at both ends of the distribution in the general population. In order to allow for more fine-grained separation at these levels of health, additional domains or items, possibly with more fine-grained response options, will be necessary to separate individuals who are relatively healthy or those that are in very poor levels of health. Finally, in the regression models, an additive effect was assumed for the different health conditions. As there is a huge number of possible interaction terms and several health conditions were rather rare, we decided not to include interaction terms to obtain a more stable and better interpretable model. Nonetheless, the same principle of constructing a metric using the approach we have used would hold true.

## Conclusions

The health metric developed in this study – based on the domains of the minimal generic set – proved useful for a wide range of health comparisons, especially for different groups of persons, and both cross-sectionally and over time. Monitoring health over time provides especially useful information for health care providers and health policymakers and both in clinical settings and the general population. Such a health metric offers a wide range of applications, including comparisons of levels of health among different groups in the general population, clinical populations, and even populations within and across different countries. Yet, for different countries and settings, the metric must be systematically evaluated.

## Additional files

Additional file 1: Questions used to operationalize the six domains of the minimal generic set of functioning and health. Questions used to operationalize the six domains of the minimal generic set and items created based on these questions. When more than one question was used to create an item, the name of the created summary measure is provided. (DOC 42 kb) Additional file 2: Item locations, item thresholds, and outfit and infit mean squares (DOC 40 kb) Additional file 3: Graphic assessment of item fit. Comparison of expected probabilities of responding above the threshold based on the PCM (red line) and observed response frequencies for groups of persons with close ability estimates (the “x”s are connected by dotted black lines). (PDF 33 kb) Additional file 4: Boxplots on convergent and discriminant validity. Boxplots of the health metric by general health, long-standing illness, life satisfaction, grouped number of falls, and age groups. Boxes are drawn with widths proportional to the square-roots of the number of observations in the groups, i.e., the smaller the box, the smaller the group size. Groups with a very small number of observations were merged. (PDF 9 kb) Additional file 5: Correlation matrix on convergent and discriminant validity. Spearman correlation matrix for the health metric and additional health-related variables for wave-4 data. General health and long-standing illness are more related to health (relevant for convergent validity), while life satisfaction, the number of falls, and age are less related to health (relevant for discriminant validity). (DOC 31 kb) Additional file 6: Results on sensitivity to change – nonlinear effect of age. Nonlinear effect of age (solid line) and 95 % confidence intervals (dashed lines) resulting from the linear additive model predicting the value of the health metric in wave 4 based on the incidence of health conditions within the last two years, when controlling for the value of the health metric in wave 3 and other covariates. (PDF 5 kb)
