# Supplementary material for: Development of a metric for tracking and comparing population health based on the minimal generic set of domains of functioning and health
Source: Popul Health Metr. 2016 May 12;14:19. doi: 10.1186/s12963-016-0088-y (PMC4866300; doi:10.1186/s12963-016-0088-y)
Supplement: Additional file 1: — Questions used to operationalize the six domains of the minimal generic set of functioning and health. Questions used to operationalize the six domains of the minimal generic set and items created based on these questions. When more than one question was used to create an item, the name of the created summary measure is provided. (DOC 42 kb) [file 12963_2016_88_MOESM1_ESM.doc]

### Additional File 1: Questions used to operationalize the six domains of the minimal generic set of functioning and health

Questions used to operationalize the six domains of the minimal generic set and items created based on these questions. When more than one question was used to create an item, the name of the created summary measure is provided.

| **Domain** | **Question** | **Item** |
| --- | --- | --- |
| Energy and drive functions | (Much of the time during the past week), you **felt** that **everything** you did **was an effort**? | 1) |
|  | (Much of the time during the past week), you **could not get going**? | 2) |
|  | *Here is a list of statements that people have used to describe their lives or how they feel. How often do you feel like this?*  - I **feel full of energy** these days | 3) |
| Emotional functions | (Much of the time during the past week), you **felt depressed**? | 4) |
|  | (Much of the time during the past week), you **felt sad**? | 5) |
|  | (Much of the time during the past week), you **were happy**? | 6) |
| Sensation of pain | Are you often troubled with pain? | 7) **Sensation of pain** |
|  | How bad is the pain most of the time? Is it mild, moderate or severe? |  |
| Carrying out daily routine | *Please tell me if you have any difficulty with these because of a physical, mental, emotional or memory problem. Again exclude any difficulties you expect to last less than three months. Because of a health or memory problem, do you have difficulty doing any of the activities on this card? -* |  |
|  | Dressing, including putting on shoes and socks | 8) **Score ADL** |
|  | Bathing or showering |  |
|  | Eating, such as cutting up food |  |
|  | Getting in or out of bed |  |
|  | Using the toilet, including getting up or sitting down |  |
|  | Using a map to figure out how to get around in a strange place | 9) **Score IADL** |
|  | Preparing a hot meal |  |
|  | Shopping for groceries |  |
|  | Taking medications |  |
|  | Doing work around the house or garden |  |
|  | Managing money, such as paying bills and keeping track of expenses |  |
| Walking and moving around | By and without using any special equipment, how much difficulty do you have **walking** for **a quarter of a mile**? | 10) |
|  | *Because of a physical or health problem, do you have difficulty doing any of the activities on this card? Exclude any difficulties that you expect to last less than three months.*  - **Walking 100 yards**. | 11) |
| Remunerative employment | Do you have any health problem or disability that limits the kind or amount of **paid work** you could do, should you want to? | 12) |
