# Supplementary material for: Development of a metric for tracking and comparing population health based on the minimal generic set of domains of functioning and health
Source: Popul Health Metr. 2016 May 12;14:19. doi: 10.1186/s12963-016-0088-y (PMC4866300; doi:10.1186/s12963-016-0088-y)
Supplement: Additional file 2: — Item locations, item thresholds, and outfit and infit mean squares (DOC 40 kb) [file 12963_2016_88_MOESM2_ESM.doc]

### Additional File 2: Item locations, item thresholds, and outfit and infit mean squares

| **Item** | **Location** | **Threshold 1** | **Threshold 2** | **Threshold 3** | **Outfit mean squares** | **Infit mean squares** |
| --- | --- | --- | --- | --- | --- | --- |
| Felt everything was an effort | 0.270 | 0.270 |  |  | 0.665 | 0.837 |
| Felt could not get going | 0.276 | 0.276 |  |  | 0.757 | 0.923 |
| Felt full of energy | -0.853 | -3.429 | -0.359 | 1.227 | 0.830 | 0.851 |
| Felt depressed | 0.624 | 0.624 |  |  | 0.727 | 0.940 |
| Felt sad | 0.189 | 0.189 |  |  | 1.148 | 1.154 |
| Was (un)happy | 1.173 | 1.173 |  |  | 0.907 | 1.037 |
| Sensation of pain | 0.403 | -0.772 | 1.579 |  | 1.176 | 1.213 |
| Score ADL | 0.981 | 0.628 | 1.333 |  | 0.583 | 0.730 |
| Score IADL | 1.074 | 0.734 | 1.414 |  | 0.590 | 0.723 |
| Walking a quarter of a mile | 0.480 | 0.127 | 0.833 |  | 0.603 | 0.765 |
| Walking 100 yards | 1.163 | 1.163 |  |  | 0.374 | 0.697 |
| Remunerative employment | -0.513 | -0.513 |  |  | 0.721 | 0.831 |
