# Supplementary figures and images for: Development of a metric for tracking and comparing population health based on the minimal generic set of domains of functioning and health
Source: Popul Health Metr. 2016 May 12;14:19. doi: 10.1186/s12963-016-0088-y (PMC4866300; doi:10.1186/s12963-016-0088-y)

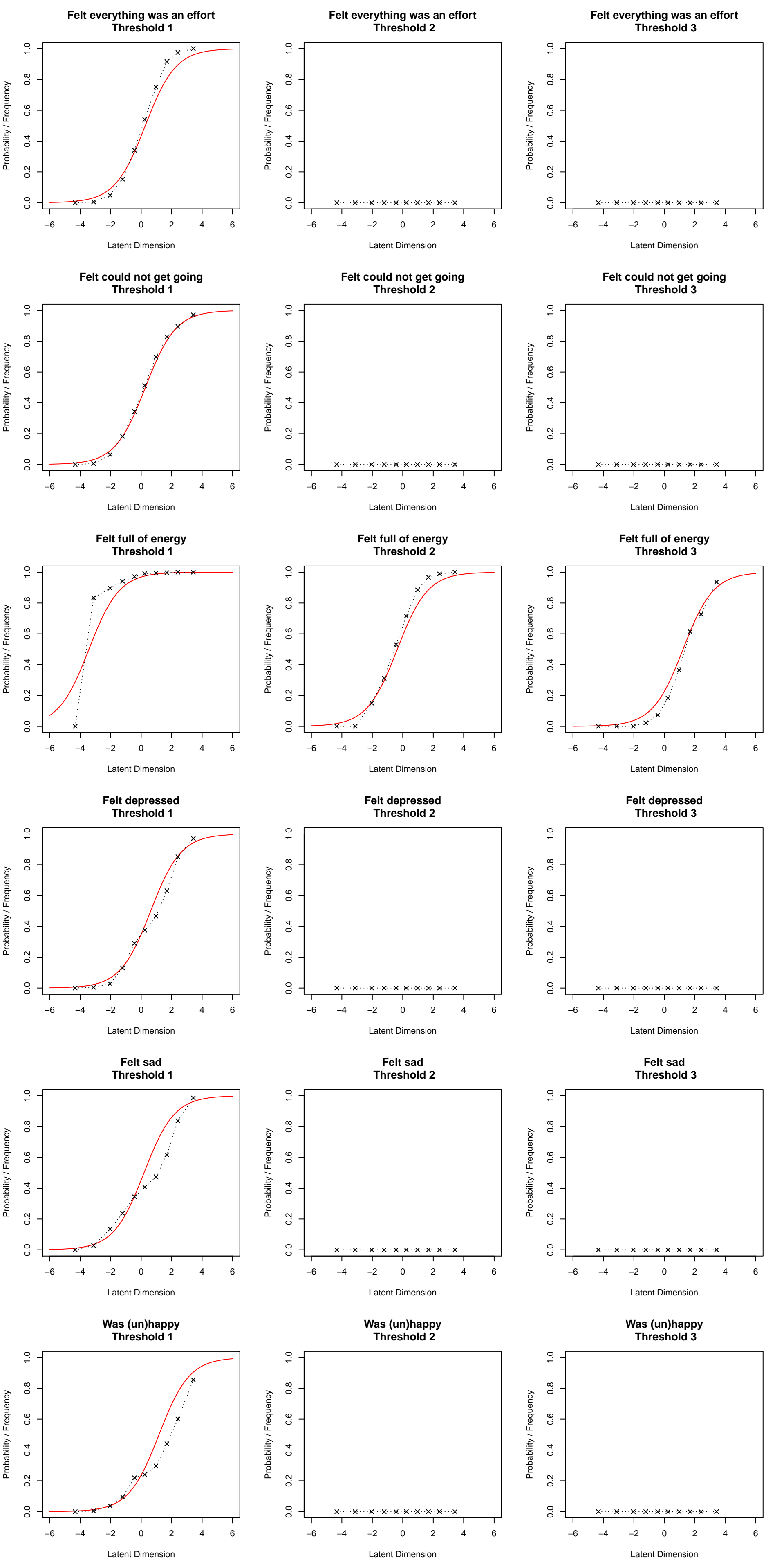

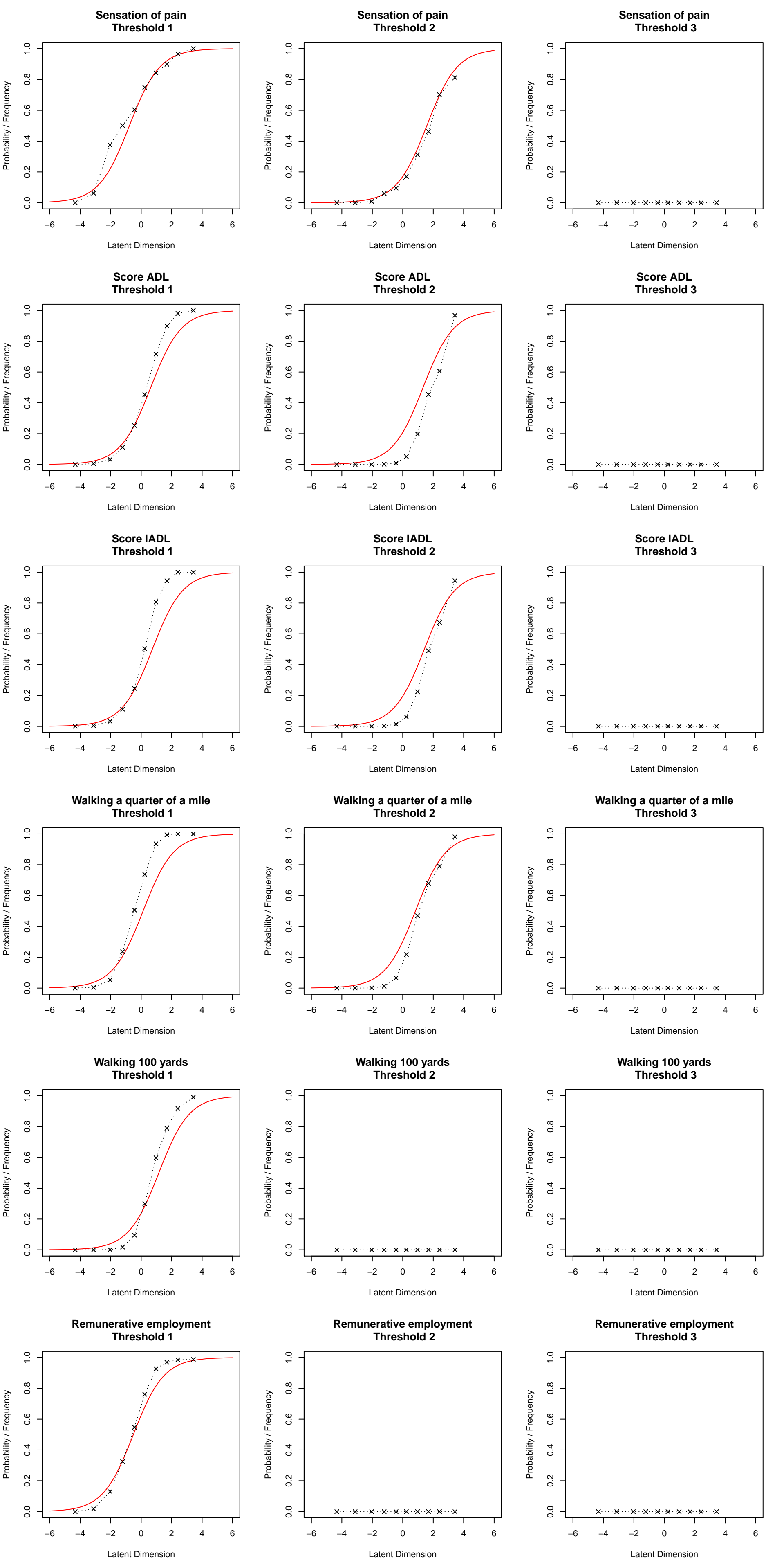

Supplement: Additional file 3: — Graphic assessment of item fit. Comparison of expected probabilities of responding above the threshold based on the PCM (red line) and observed response frequencies for groups of persons with close ability estimates (the “x”s are connected by dotted black lines). (PDF 33 kb) [file 12963_2016_88_MOESM3_ESM.pdf]

General Health

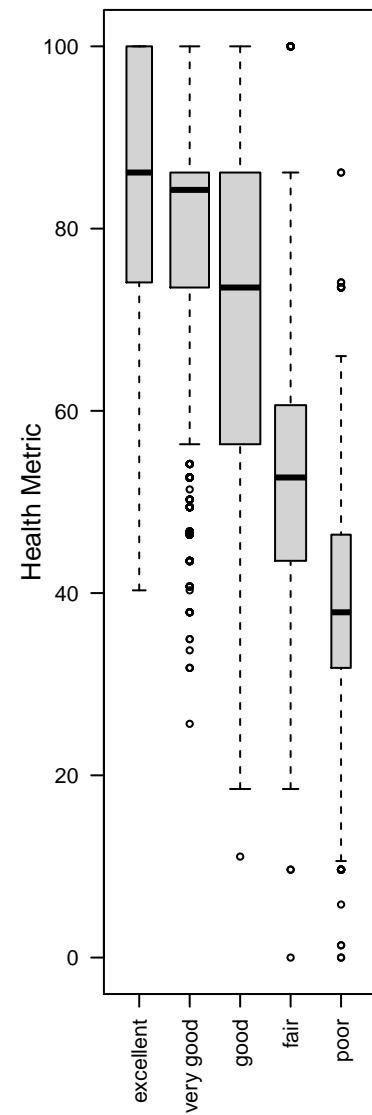

Long-standing Illness

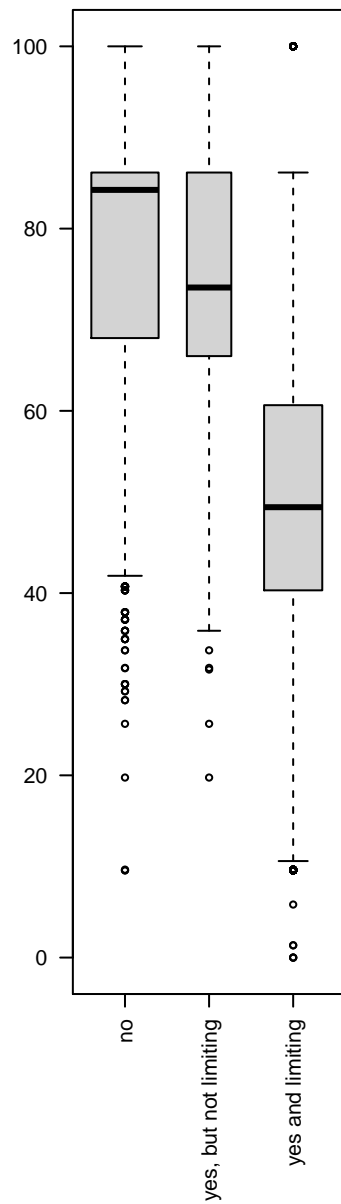

Life Satisfaction

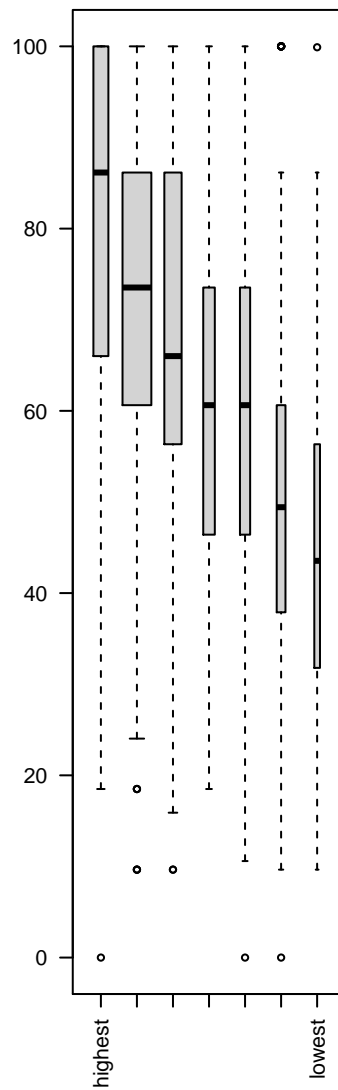

Number of Falls

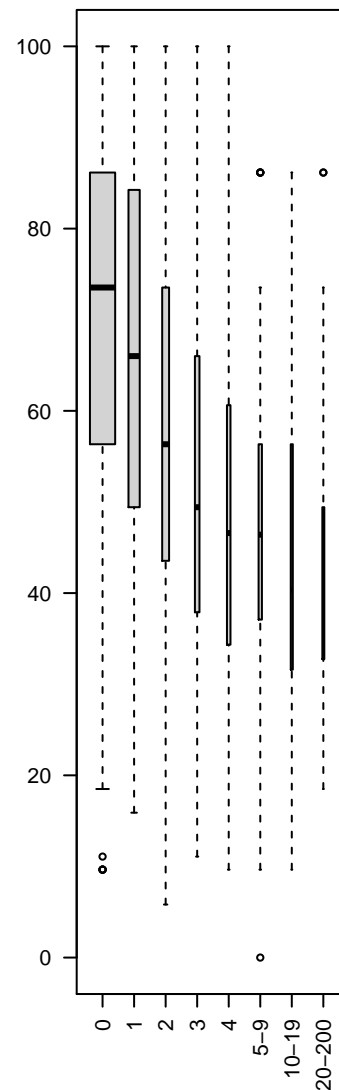

Age

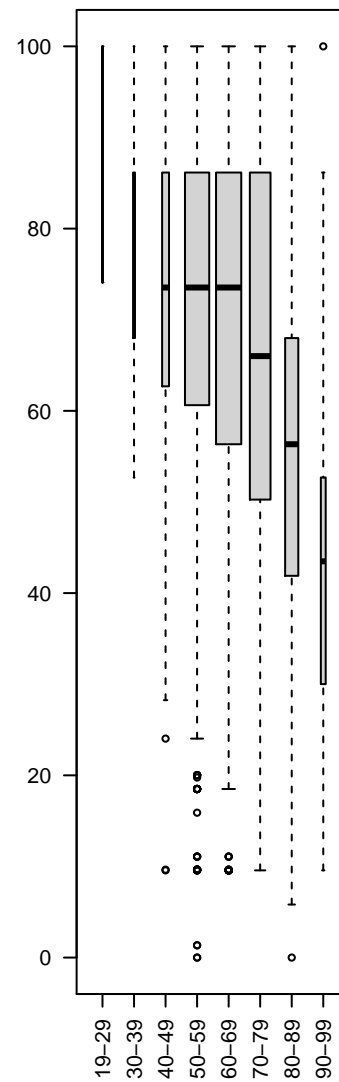

Supplement: Additional file 4: — Boxplots on convergent and discriminant validity. Boxplots of the health metric by general health, long-standing illness, life satisfaction, grouped number of falls, and age groups. Boxes are drawn with widths proportional to the square-roots of the number of observations in the groups, i.e., the smaller the box, the smaller the group size. Groups with a very small number of observations were merged. (PDF 9 kb) [file 12963_2016_88_MOESM4_ESM.pdf]

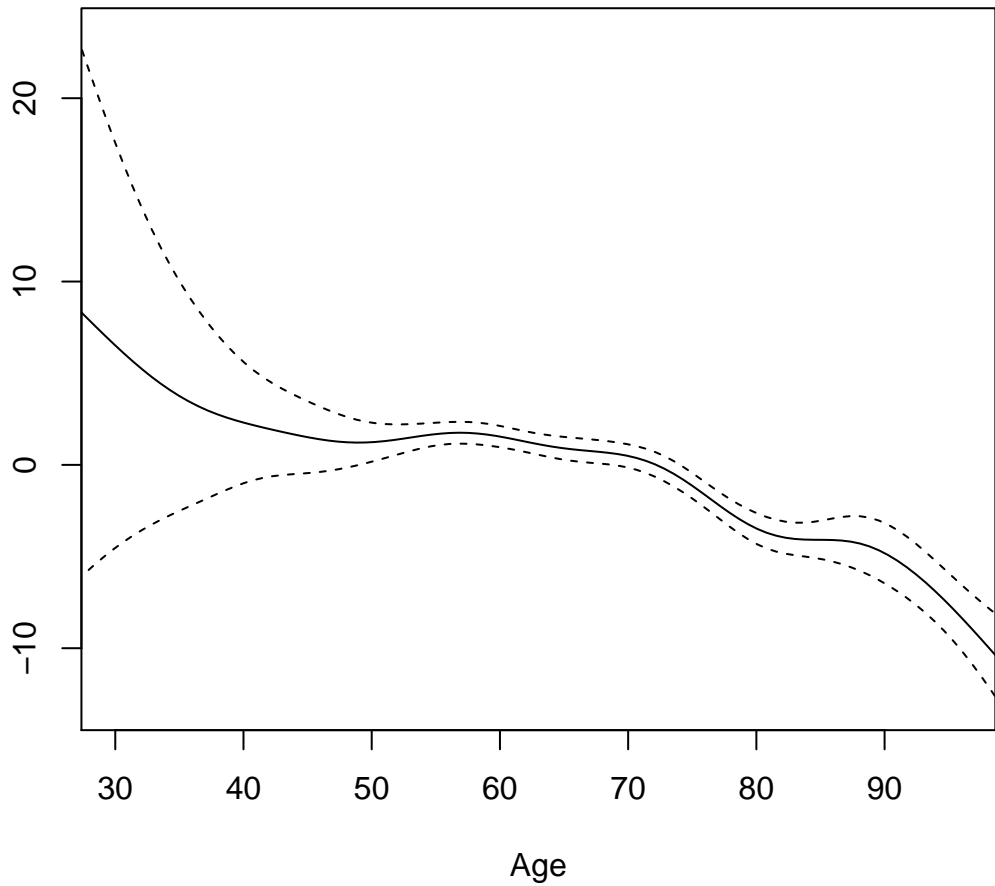

Supplement: Additional file 6: — Results on sensitivity to change – nonlinear effect of age. Nonlinear effect of age (solid line) and 95 % confidence intervals (dashed lines) resulting from the linear additive model predicting the value of the health metric in wave 4 based on the incidence of health conditions within the last two years, when controlling for the value of the health metric in wave 3 and other covariates. (PDF 5 kb) [file 12963_2016_88_MOESM6_ESM.pdf]
