# Supplementary material for: Development of a metric for tracking and comparing population health based on the minimal generic set of domains of functioning and health
Source: Popul Health Metr. 2016 May 12;14:19. doi: 10.1186/s12963-016-0088-y (PMC4866300; doi:10.1186/s12963-016-0088-y)
Supplement: Additional file 5: — Correlation matrix on convergent and discriminant validity. Spearman correlation matrix for the health metric and additional health-related variables for wave-4 data. General health and long-standing illness are more related to health (relevant for convergent validity), while life satisfaction, the number of falls, and age are less related to health (relevant for discriminant validity). (DOC 31 kb) [file 12963_2016_88_MOESM5_ESM.doc]

### Additional File 5: Correlation matrix on convergent and discriminant validity

Spearman correlation matrix for the health metric and additional health-related variables for wave-4 data. General health and long-standing illness are more related to health (relevant for convergent validity), while life satisfaction, the number of falls, and age are less related to health (relevant for discriminant validity). The intensity of the cell color visualizes the degree of correlation. The darker the cell, the higher the correlation.

|  | **Health metric** | **General health** | **Long-standing illness** | **Life satisfaction** | **Number of falls** | **Age** |
| --- | --- | --- | --- | --- | --- | --- |
| Health metric | 1.00 | -0.64 | -0.59 | -0.36 | -0.25 | -0.23 |
| General health ^#^ | -0.64 | 1.00 | 0.56 | 0.26 | 0.17 | 0.19 |
| Long-standing illness ^*^ | -0.59 | 0.56 | 1.00 | 0.18 | 0.19 | 0.19 |
| Life satisfaction ^+^ | -0.36 | 0.26 | 0.18 | 1.00 | 0.08 | -0.06 |
| Number of falls | -0.25 | 0.17 | 0.19 | 0.08 | 1.00 | 0.10 |
| Age | -0.23 | 0.19 | 0.19 | -0.06 | 0.10 | 1.00 |

^#^ General health: self-rated general health question with five response options from “excellent” to “poor”
^*^ Long-standing illness: long-standing limiting illness, disability or infirmity (response options: “no,” “yes, but not limiting,” “yes and limiting”)
^+^ Life satisfaction measured with seven response options
